# Internet-Based Cognitive Behavioral Therapy via Videoconference for Patients With Bulimia Nervosa and Binge-Eating Disorder: Pilot Prospective Single-Arm Feasibility Trial

**DOI:** 10.2196/15738

**Published:** 2019-10-23

**Authors:** Sayo Hamatani, Noriko Numata, Kazuki Matsumoto, Chihiro Sutoh, Hanae Ibuki, Keiko Oshiro, Mari Tanaka, Rikukage Setsu, Yohei Kawasaki, Yoshiyuki Hirano, Eiji Shimizu

**Affiliations:** 1 Research Center for Child Mental Development Chiba University Chiba Japan; 2 Japan Society for the Promotion of Science Tokyo Japan; 3 Department of Cognitive Behavioral Physiology Graduate School of Medicine Chiba University Chiba Japan; 4 Koutokukai Sato Hospital Yamagata Japan; 5 Biostatistics Section Clinical Research Center Chiba University Hospital Chiba Japan; 6 Cognitive Behavioral Therapy Center Chiba University Hospital Chiba Japan

**Keywords:** bulimia nervosa, binge-eating disorder, cognitive behavioral therapy, internet-based cognitive behavioral therapy, videoconference

## Abstract

**Background:**

A major problem in providing mental health services is the lack of access to treatment, especially in remote areas. Thus far, no clinical studies have demonstrated the feasibility of internet-based cognitive behavioral therapy (ICBT) with real-time therapist support via videoconference for bulimia nervosa and binge-eating disorder in Japan.

**Objective:**

The goal of the research was to evaluate the feasibility of ICBT via videoconference for patients with bulimia nervosa or binge-eating disorder.

**Methods:**

Seven Japanese subjects (mean age 31.9 [SD 7.9] years) with bulimia nervosa and binge-eating disorder received 16 weekly sessions of individualized ICBT via videoconference with real-time therapist support. Treatment included CBT tailored specifically to the presenting diagnosis. The primary outcome was a reduction in the Eating Disorder Examination Edition 16.0D (EDE 16D) for bulimia nervosa and binge-eating disorder: the combined objective binge and purging episodes, objective binge episodes, and purging episodes. The secondary outcomes were the Eating Disorders Examination Questionnaire, Bulimic Investigatory Test, Edinburgh, body mass index for eating symptoms, Motivational Ruler for motivation to change, EuroQol-5 Dimension for quality of life, 9-item Patient Health Questionnaire for depression, 7-item Generalized Anxiety Disorder scale for anxiety, and Working Alliance Inventory–Short Form (WAI-SF). All outcomes were assessed at week 1 (baseline) and weeks 8 (midintervention) and 16 (postintervention) during therapy. Patients were asked about adverse events at each session. For the primary analysis, treatment-related changes were assessed by comparing participant scores and 95% confidence intervals using the paired *t* test.

**Results:**

Although the mean combined objective binge and purging episodes improved from 47.60 to 13.60 (71% reduction) and showed a medium effect size (Cohen *d*=–0.76), there was no significant reduction in the combined episodes (EDE 16D –41; 95% CI –2.089 to 0.576; *P*=.17). There were no significant treatment-related changes in secondary outcomes. The WAI-SF scores remained consistently high (64.8 to 66.0) during treatment.

**Conclusions:**

ICBT via videoconference is feasible in Japanese patients with bulimia nervosa and binge-eating disorder.

**Trial Registration:**

UMIN Clinical Trials Registry UMIN000029426; https://upload.umin.ac.jp/cgi-open-bin/ctr_e/ctr_view.cgi?recptno=R000033419

## Introduction

Eating disorders characterized by hyperphagia include bulimia nervosa and binge-eating disorder [1]. The prevalence of bulimia nervosa in Japan is estimated to be 1.9% to 2.9% [2]. Bulimia nervosa is a psychiatric disorder characterized by repeated overeating and inappropriate compensatory behavior [1]. Like bulimia nervosa, binge-eating disorder is characterized by repeated episodes of hyperphagia but without the repetitive inappropriate compensatory behavior seen in bulimia nervosa [1]. Several meta-analyses of randomized controlled trials (RCTs) that included wait list controls have reported that cognitive behavioral therapy (CBT) is effective for bulimia nervosa and binge-eating disorder [3,4-7]. Our research group has recently conducted a single-arm study that confirmed the effectiveness of guided self-help CBT for Japanese patients with bulimia nervosa [8]. Although previous studies have shown the effectiveness of CBT for bulimia nervosa and binge-eating disorder, the patients who reside in rural areas or far from specialized treatment institutions cannot receive evidence-based therapy such as CBT. Comorbidities such as panic disorder and agoraphobia make it difficult for patients to go out, and patients with severe depression are easily tired, making it difficult for patients to attend CBT sessions once a week. Therefore, it is necessary to devise methods to provide the approved treatment at home for all patients.

Recently, CBT for patients with eating disorders has been increasingly provided via the internet [9-13]. Most internet-based CBT (ICBT) clinical trials provided to patients are guided by the therapist via email, but not in real time. A systematic review of 8 studies in patients with bulimia nervosa, binge-eating disorder, or eating disorder not otherwise specified (EDNOS) suggested that ICBT without videoconference has the effect of reducing the frequency of overeating [14]. Further, a systematic review including 22 RCTs, 2 controlled studies, and 16 uncontrolled studies found evidence for the efficacy of guided ICBT for bulimia nervosa patients; however, only one RCT included ICBT via videoconference [15]. Hence, more research is needed to determine the efficacy and limitations of ICBT via videoconference. There have been 2 case series and one clinical trial of ICBT via videoconference reported specifically in patients with eating disorders [16-18]. A case series with ICBT via videoconference was performed on two patients with bulimia nervosa in the United States [16]. A case series of ICBT via videoconference was conducted in Scotland and included 12 patients: bulimia nervosa (n=5), anorexia nervosa (n=1), and EDNOS (n=6, including 3 with binge-eating disorder) [17]. A total of 67% of these patients rated ICBT via videoconference favorably because of the convenience of receiving treatment in their area, feeling more in control, and feeling less intimidated than in a face-to-face session. An RCT was performed in the United States and included 128 patients with bulimia nervosa and EDNOS who were assigned to face-to-face CBT or ICBT via videoconference [18]. The posttreatment symptom rating scale score and symptomatic improvement showed no significant difference between ICBT via videoconference and face-to-face CBT.

The evidence for the effectiveness of ICBT via videoconference for bulimia nervosa and binge-eating disorder appears promising but is limited and has only been demonstrated in Western populations. No clinical trials of ICBT via videoconference have been conducted in non-Western populations, including in Japan. The aim of this study was to evaluate the feasibility of ICBT via videoconference for Japanese patients with bulimia nervosa and binge-eating disorder by evaluating the posttreatment change in symptom evaluation scores.

## Methods

### Study Design

This prospective single-arm open trial was performed in the outpatient clinic at the Cognitive Behavioral Therapy Center of Chiba University Hospital between October 2017 and March 2019. Because of the deadline for grant support, we could not extend the recruitment period. Since this trial was the first to employ an individual ICBT via videoconference intervention design against bulimia nervosa and binge-eating disorder in Japan, a single-arm trial examining feasibility rather than effectiveness was considered to be an appropriate design.

### Ethics Statement

The study was approved by the institutional review board of Chiba University Hospital (reference number G29028) and registered at University Hospital Medical Information Network Clinical Trials Registry [UMIN000029426]. Written informed consent was obtained from all patients who participated in the study after they had been fully informed about the study protocol.

### Study Participants

Patients were enrolled in the study if they met the following inclusion criteria: assessment by a senior psychiatrist at Chiba University Hospital using the Mini International Neuropsychiatric Interview [19,20] and a primary diagnosis of bulimia nervosa or binge-eating disorder according to the *Diagnostic and Statistical Manual of Mental Disorders, 5th Edition* (DSM-5) criteria, females aged 16 to 65 years; BMI >17.5 kg/m^2^, and access to the internet at home. A comorbid diagnosis was accepted if it was clearly secondary to bulimia nervosa and binge-eating disorder. Exclusion criteria involved those who were expected to interrupt CBT due to the following comorbidities: organic brain damage, dementia, psychotic disorders, bipolar disorder, current high risk of suicide, substance abuse or dependence, antisocial behaviors, and critical physical disease. In principle, no drug changes were allowed during the trial.

### Cognitive Behavioral Therapists and Quality Control

A total of 16 one-on-one sessions were conducted between therapists and patients. The therapists were three clinical psychologists and one nurse; all therapists were certified public psychologists. All therapists had completed a CBT training course (ie, Chiba–Improving Access to Psychological Therapies project) [21].

### Primary Outcome

The primary outcome was the change from week 1 (baseline) to week 16 (postintervention) in frequency of objective binge and purging episodes assessed by the Eating Disorder Examination Edition 16.0D (EDE 16D) [22,23]. It contains subscales that reflect the frequency of episodes of a particular behavior, frequency of days on which the behavior occurred, and psychopathologic severity of the eating disorder. In this study, we used only the frequency of episodes of behavior, which is an important feature of eating disorders. The frequency of objective binge episodes over 28 days, frequency of purging episodes (vomiting, laxative abuse, and diuretic abuse), and frequency of combined objective binge and purging episodes were calculated. Each subject was assessed at week 1 (baseline) and week 16 (postintervention).

### Secondary Outcomes

Each patient was also assessed using the Eating Disorders Examination Questionnaire (EDE-Q) [23,24], Bulimic Investigatory Test, Edinburgh (BITE) [25,26], BMI, Patient Health Questionnaire (PHQ-9) [27,28], Generalized Anxiety Disorder scale (GAD-7) [29,30], EuroQol-5 Dimensions (EQ-5D-5L) [31,32], Working Alliance Inventory-Short Form (WAI-SF) [33], and Motivational Ruler [34] at weeks 1 (baseline), 8 (midintervention), and 16 (postintervention).

### Psychopathologic Severity of Bulimia Nervosa or Binge-Eating Disorder

The psychopathologic severity of bulimia nervosa or binge-eating disorder was assessed using the EDE-Q [23,24], which is a self-contained, 28-item questionnaire derived from the EDE. The EDE-Q is scored on a 7-point Likert scale (0 to 6) on which a score of ≥4 indicates a clinical range. The global score on the EDE-Q is the sum of the 4 subscale scores (restraint, eating concern, shape concern, and weight concern) divided by 4. The presence and severity of symptoms of bulimia nervosa and cognitive and emotional signs and symptoms associated with hyperphagia were assessed using the BITE [25,26]. The BITE is a self-administered 33-item questionnaire that consists of 30 symptom subscales (eg, “Would you say that food dominated your life?”) and 3 severity subscales (“I don't eat all day,” “how to lose weight,” and “frequency of overeating”). Symptom subscale items are organized in yes/no format, and severity subscale items are organized in a Likert-type response format (5-point or 7-point, depending on the item). The symptom subscale has a minimum score of 0 and a maximum score of 30. However, the maximum severity subscale score is 39. The symptom subscale has 20 or more points ranging from “altered pattern of behavior” to “high probability of bulimia nervosa.” A severity subscale score of ≥5 indicates a clinically significant eating disorder.

### Depression and Generalized Anxiety

The presence and severity of symptoms of depression experienced in the previous 2 weeks was evaluated using the PHQ-9 [27,28]. The self-administered questionnaire items are scored on a 4-point Likert-scale (0=not at all, 1=on several days, 2=half or more of the days, 3=almost every day). The minimum score is 0 and the maximum score is 27 (0 to 4 indicates no symptoms, 5 to 9 indicates mild symptoms, 10 to 14 indicates moderate symptoms, 15 to 19 indicates moderate to severe symptoms, and 20 to 27 indicates severe symptoms). The cutoff score for clinically significant depressive symptoms is 10. The presence and severity of generalized anxiety disorder was assessed using the GAD-7 [29,30], a self-administered questionnaire that assesses the severity of generalized anxiety disorder in the previous 2 weeks on a 4-point Likert scale (0=not at all, 1=one episode, 2=on half or more days, 3=almost every day). The minimum score is 0 and maximum score is 21 (0 to 4 indicates no symptoms, 5 to 9 indicates mild symptoms, 10 to 14 indicates moderate symptoms, and 15 to 21 indicates severe symptoms). The cutoff score for clinically significant symptoms of anxiety is 10.

### Quality of Life

Quality of life was measured using the EQ-5D-5L questionnaire [31,32]. The EQ-5D-5L is self-administered with items that are scored from 0 (death) to 1 (in good health).

### Therapeutic Relationship and Motivation

This study was the first to use ICBT via videoconference to provide therapist support for Japanese patients with bulimia nervosa and binge-eating disorder. ICBT via videoconferencing used the following scales to determine whether motivation and treatment compliance could be established. The strength of the therapeutic alliance was assessed using the WAI-SF [33]. This self-administered questionnaire consists of 12 items that measure 3 factors (ie, agreement on the task of treatment, agreement on the goal of treatment, and a bond between the therapist and patient). The WAI-SF is scored on a 7-point Likert scale; the minimum score is 12, and the maximum is 84. Motivation for treatment was assessed using the Motivational Ruler [34]. This instrument is a self-administered questionnaire containing 2 items (“How important is it for you to change and recover from your eating disorder?” and “How confident are you in your ability to change and recover from your eating disorder?”) that are measured on a 10-point Likert scale. The minimum score is 2, and the maximum score is 20.

### Interventions

Participants entered the Web conference room by clicking on the URL in the email sent by the therapist. The intervention was performed for 50 minutes once a week for 16 weeks. The protocol included the following modules: assessment; explanation of CBT, motivation and goal setting, education on bulimia nervosa and how to keep a dietary diary, psychological education and monitoring for overeating behavior, psychological education and monitoring of diet, reforming of dietary habits (mindful diet), psychological education and self-monitoring of compensatory behavior, stress management and relaxation, understanding perfectionism, interpersonal relationships and assertions, acquisition of skills to associate with emotions (emotional adjustment, mindfulness, tolerance of distress, alexithymia), attention to body image, and prevention of recurrence. These modules are a development of those used in our previous study of face-to-face CBT for patients with bulimia nervosa in Japan [8,35].

### Visual Aids

The use of visual aids promotes the learning process by enhancing motivation and understanding of complex concepts [36]. To deepen understanding, therapists presented a visual aid that consisted of several slides containing the key concepts of CBT using the screen-sharing feature of the videoconferencing software. After each session, study participants were emailed a set of password-protected homework slides.

### Hardware

The therapists used a Surface Pro 2 computer (Microsoft Corp), which is a 2-in-1 detachable system that runs on Windows 10 Pro (Microsoft Corp). This computer has a display size of 10.6 inches and a resolution of 1920×1080 pixels.

### Videoconferencing Software

A total of 3 licenses for videoconferencing software (Cisco Webex, Cisco Systems) were used. This system has International Organization for Standardization 27001 certification (for handling information security) and Statement on Standards for Attestation Engagements Number 16–compliant certification issued by a third party. Cisco Webex’s use of a switching network with 128-bit Secure Sockets Layer encryption and public key infrastructure is considered to have resolved the problem of security [37]. We determined that Cisco Webex can be trusted for the purposes of this research because the software is stable and secure and protects personal information well.

### Adverse Events

At the end of each session, therapists asked patients about their overall physical and mental state of health and instructed them to report any adverse events experienced after the intervention by email. No mental or physical adverse events were reported.

### Statistical Analysis

Statistical analysis and reporting of this trial were conducted in accordance with the Consolidated Standards of Reporting Trials of Electronic and Mobile Health Applications and Online TeleHealth (CONSORT-EHEALTH) guidelines [38]. The primary analysis compared differences in objective binge and purging on the EDE 16D between baseline and 16 weeks for all patients with bulimia nervosa or binge-eating disorder using the paired *t* test, and 95% confidence intervals were calculated. Analysis of secondary outcomes was performed in the same way. We also calculated the effect size of treatment using Cohen *d*, calculated as the mean difference after treatment divided by the pooled standard deviation. A Cohen *d*>0.20 was used as the criterion for a small effect, a value >0.50 as a medium effect, and >0.80 as a large effect [39]. All *P* values were 2-sided and those <.05 were considered statistically significant. All statistical analyses were performed with SAS software version 9.4 (SAS Institute Inc).

## Results

### Recruitment

**Figure 1:**
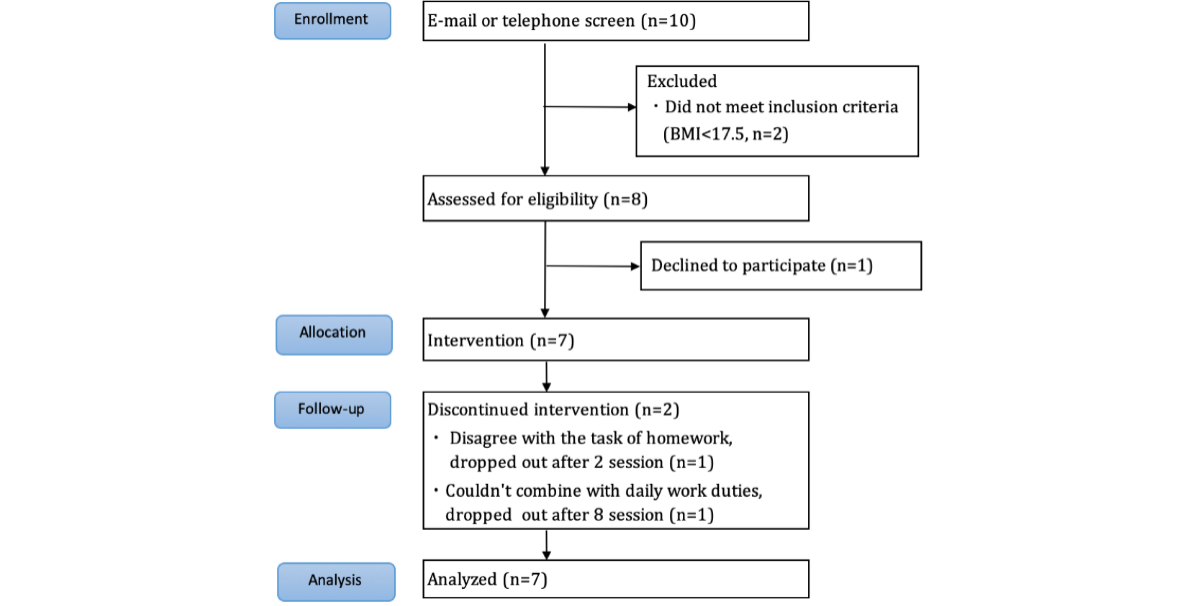
Flow of participants.

Figure 1 shows the flow of participants through the study. Ten patients applied to participate in the study via our website. After screening by email and telephone, 2 patients failed to meet the inclusion criteria and were excluded (BMI <17.5 kg/m^2^); 8 patients attended a baseline face-to-face assessment and were enrolled in the study. After enrollment, 1 patient declined to participate, and 2 patients dropped out during the intervention (1 refused to perform the homework; 1 could not combine the task assigned with her daily work). The analysis included the data of these 2 patients.

### Demographic and Clinical Characteristics

The study population comprised 7 women of mean age 31.9 (SD 7.9; range 21-43) years. Their demographic and clinical characteristics are shown in Table 1. Five participants continued to receive pharmacotherapy during the trial (triazolam, n=1; flunitrazepam, n=1; clonazepam, n=1; sertraline, n=1; sodium valproate, n=1; quetiapine fumarate, n=1; aripiprazole, n=1; and clotiazepam, n=1).

**Table 1:** Clinical and demographic characteristics of the study participants (n=7).

Characteristic		Participant							Value
		1	2	3	4	5	6	7	
Sex		Female	Female	Female	Female	Female	Female	Female	—
Age, mean (SD)		26	43	31	29	43	24	27	31.9 (7.9)
Education in years, mean (SD)		14	15	17	11	12	12	16	14.1 (2.4)
Duration of eating disorder in years, mean (SD)		12	17	17	12	19	5	2	12.0 (6.4)
BMI (kg/m^2^), mean (SD)		17.8	22.2	20.4	18.4	36.0	20.0	30.0	23.5 (6.8)
Diagnosis		Bulimia nervosa	Bulimia nervosa	Bulimia nervosa	Bulimia nervosa	Binge-eating disorder	Bulimia nervosa	Binge-eating disorder	—
**Comorbidity, n (%)**									
	Major depressive disorder	—	—	—	X	X	—	—	2 (29)
	Dysthymia	—	—	—	X	—	—	X	2 (29)
	Agoraphobia	—	—	—	—	X	—	—	1 (14)
	Social anxiety disorder	X	—	—	—	—	—	X	2 (29)
	Obsessive-compulsive disorder	—	—	X	—	—	—	—	1 (14)
	Posttraumatic stress disorder	—	—	X	—	—	X	—	1 (14)
	Generalized anxiety disorder	—	—	—	X	—	X	—	2 (29)
	Schizophrenia	—	—	—	—	—	—	X	1 (14)
**Past history, n (%)**									
	Major depressive disorder	—	—	—	—	X	—	—	1 (14)
	Manic episode	—	—	—	—	X	—	X	2 (29)
Autism spectrum quotient, mean (SD)		28	13	15	33	33	19	17	22.6 (7.9)
Estimated IQ by JART^a^, mean (SD)		106	106	98	89	94	100	106	99.9 (6.2)
Psychotropic drug, n (%)		—	—	X	X	X	X	X	5 (71)
Completer, n (%)		X	X	X	X	X	—	—	5 (71)

^a^JART: Japanese Adult Reading Test.

### Primary Outcome

**Figure 2:**
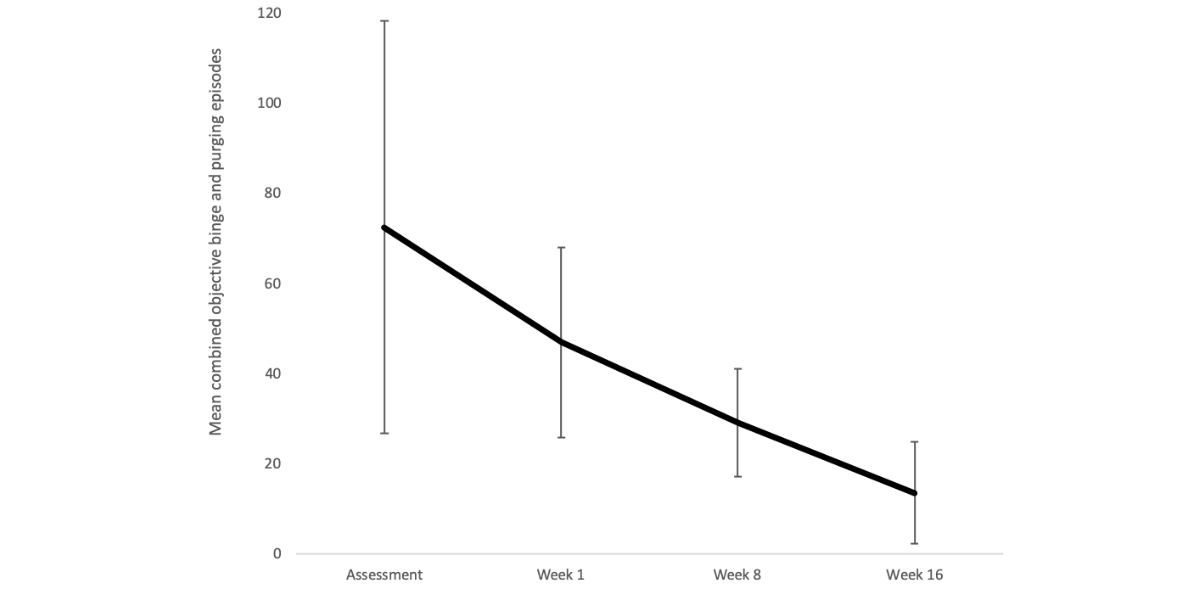
Changes in frequency of objective binge and purging episodes during treatment. Bars indicate 1 standard deviation.

**Figure 3:**
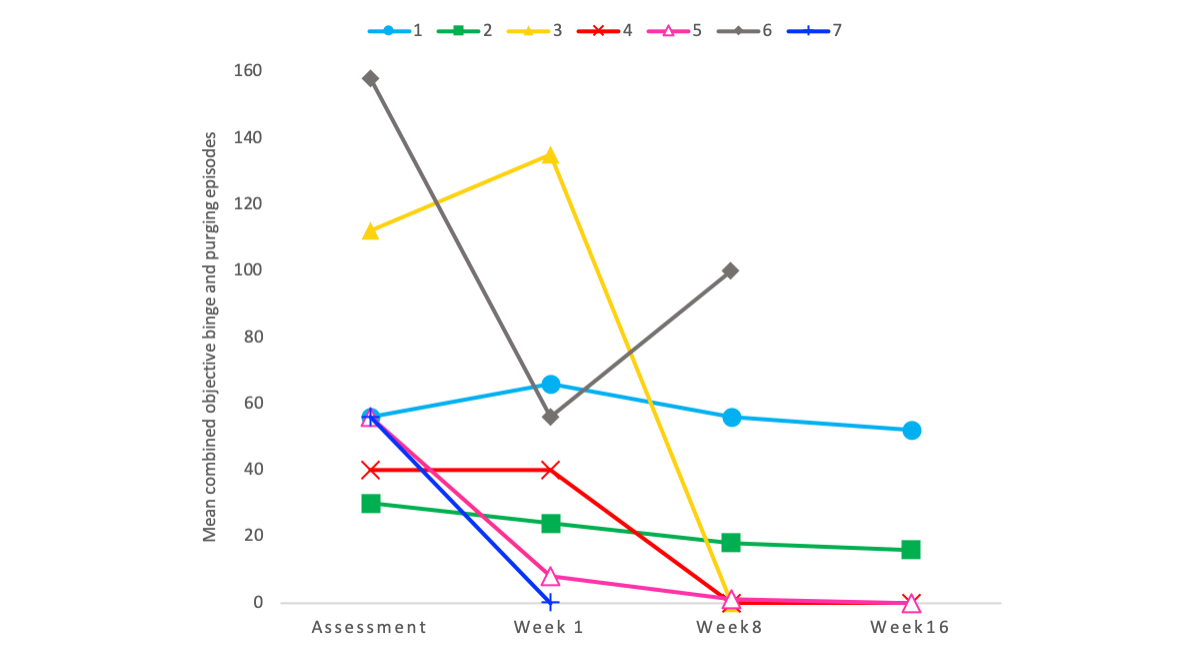
Change in number of episodes during treatment.

Figure 2 shows the change in the combined objective binge and purging episodes on the EDE 16D, and Figure 3 shows the detailed transition of symptoms for each patient. Although the mean combined episodes improved from 47.0 to 13.6 (71% reduction) and showed a medium effect size (Cohen *d*=–0.76), there was no significant reduction in the combined objective binge and purging episodes (EDE 16D −41; 95% CI −2.09 to 0.58; *P*=.17; Table 2). The frequency of objective binge episodes improved from 23.6 to 6.8 (71% reduction) and showed a large effect size (Cohen *d*=–0.82); no significant reduction in objective binge episodes (EDE 16D –20.60; 95% CI –2.18 to 0.53; *P*=.14). The frequency of purging episodes improved from 23.4 to 6.8 (71% reduction) and showed a medium effect size (Cohen *d*=–0.70); no significant reduction in the frequency of purging episodes (EDE 16D –20.40; 95% CI 2.01 to 0.62; *P*=.19). Additionally, abstinence was defined as none of these behaviors reported in the previous 28 days [18]. The abstinence rate from objective binge-eating, purging, and combined objective binge and purging was 43% (3/7).

### Secondary Outcomes

Table 3 shows the change in secondary outcomes during the study. There were no significant changes in results.

**Table 2:** Changes in the frequency of objective binge and purging episodes from pre- to post–internet-based cognitive behavioral therapy.

Episode and assessment time point		n	Mean (SD)	Cohen *d* (n=5)	95% CI	*P* value
**Combined objective binge eating and purging episodes**		—	—	–0.76	–2.09 to 0.58	.17
	Assessment	7	72.6 (45.7)			
	Week 1	7	47.0 (45.6)	—	—	—
	Week 8	7	29.2 (40.9)			
	Week 16	5	13.6 (22.6)	—	—	—
**Objective binge eating episodes**		—	—	–0.82	–2.18 to 0.53	.14
	Assessment	7	32.6 (15.8)			
	Week 1	7	29.2 (40.9)	—	—	—
	Week 8	6	9.7 (11.9)			
	Week 16	5	6.8 (11.3)	—	—	—
**Purging episodes**		—	—	–0.70	–2.01 to 0.62	.19
	Assessment	7	40.0 (31.5)			
	Week 1	7	13.6 (22.6)	—	—	—
	Week 8	6	19.5 (31.6)			
	Week 16	5	6.8 (11.3)	—	—	—

**Table 3:** Changes in psychological test scores from pre- and post–internet-based cognitive behavioral therapy.

Test name and assessment time point			n	Mean (SD)	Cohen *d* (n=5)	95% CI	*P* value
**EDE-Q^a^**							
	**Global score**		—	—	**–0.48**	**–1.158 to 0.204**	**.35**
		Assessment	7	3.2 (1.7)	—^b^	—	—
		Week 1	7	2.2 (1.3)	—	—	—
		Week 8	6	1.8 (1.3)	—	—	—
		Week 16	5	1.3 (1.1)	—	—	—
	**Restraint subscale**		—	—	**–0.33**	**–1.401 to 0.741**	**.50**
		Assessment	7	3.1 (1.9)	—	—	—
		Week 1	7	2.3 (1.9)	—	—	—
		Week 8	6	1.6 (1.4)	—	—	—
		Week 16	5	1.4 (0.9)	—	—	—
	**Eating concern subscale**		—	—	**–1.07**	**–1.957 to –0.179**	**.08**
		Assessment	7	3.3 (1.8)	—	—	—
		Week 1	7	1.9 (1.7)	—	—	—
		Week 8	6	1.2 (1.4)	—	—	—
		Week 16	5	0.7 (0.2)	—	—	—
	**Shape concern subscale**		—	—	**0.01**	**–0.273 to 0.297**	**.98**
		Assessment	7	3.4 (1.9)	—	—	—
		Week 1	7	2.5 (2.0)	—	—	—
		Week 8	6	2.3 (2.0)	—	—	—
		Week 16	5	1.6 (1.8)	—	—	—
	**Weight concern subscale**		—	—	**0.26**	**0.044 to 0.484**	**.59**
		Assessment	7	3.0 (2.0)	—	—	—
		Week 1	7	2.2 (2.0)	—	—	—
		Week 8	6	2.0 (1.6)	—	—	—
		Week 16	5	1.5 (1.9)	—	—	—
**BITE^c^**							
	**Severity**		—	—	**–1.04**	**–2.349 to 0.262**	**.08**
		Assessment	7	9.6 (2.8)	—	—	—
		Week 1	7	8.0 (4.0)	—	—	—
		Week 8	6	7.8 (7.7)	—	—	—
		Week 16	5	4.6 (3.4)	—	—	—
	**Symptoms**		—	—	**–1.08**	**–2.069 to –0.087**	**.07**
		Assessment	7	21.4 (4.1)	—	—	—
		Week 1	7	17.0 (3.9)	—	—	—
		Week 8	6	13.5 (4.6)	—	—	—
		Week 16	5	12.0 (6.6)	—	—	—
**BMI**			—	—	**0.40**	**0.096 to 0.708**	**.42**
	Assessment		7	23.5 (6.8)	—	—	—
	Week 1		7	22.8 (6.0)	—	—	—
	Week 8		6	22.4 (6.2)	—	—	—
	Week 16		5	23.1 (6.9)	—	—	—
**PHQ-9^d^**			—	—	**–0.35**	**–1.278 to 0.576**	**.48**
	Assessment		7	14.0 (6.5)	—	—	—
	Week 1		7	9.3 (3.8)	—	—	—
	Week 8		6	8.2 (2.7)	—	—	—
	Week 16		5	8.4 (4.8)	—	—	—
**GAD-7^e^**			—	—	**–0.52**	**–1.586 to 0.550**	**.31**
	Assessment		7	11.1 (5.7)	—	—	—
	Week 1		7	7.6 (5.1)	—	—	—
	Week 8		6	8.3 (5.6)	—	—	—
	Week 16		5	5.8 (1.5)	—	—	—
**EQ-5D^f^**			—	—	**0.46**	**–0.660 to 1.577**	**.36**
	Assessment		7	0.7 (0.1)	—	—	—
	Week 1		7	0.7 (0.2)	—	—	—
	Week 8		6	0.8 (0.2)	—	—	—
	Week 16		5	0.7 (0.1)	—	—	—
**WAI-SF^g^**			—	—	**0.13**	**–0.852 to 1.118**	**.78**
	Assessment		7	—	—	—	—
	Week 1		7	65.6 (8.4)	—	—	—
	Week 8		6	63.7 (10.5)	—	—	—
	Week 16		5	66.0 (4.4)	—	—	—
**Motivational ruler**			—	—	**0.00**	**–0.684 to 0.684**	**>.99**
	Assessment		7	15.4 (3.6)	—	—	—
	Week 1		7	16.4 (3.5)	—	—	—
	Week 8		6	18.3 (1.9)	—	—	—
	Week 16		5	16.0 (2.7)	—	—	—

^a^EDE-Q: Eating Disorder Examination Questionnaire.

^b^Not applicable.

^c^BITE: Bulimic Investigatory Test, Edinburgh.

^d^PHQ-9: 9-item Patient Health Questionnaire.

^e^GAD-7: 7-item Generalized Anxiety Disorder scale.

^f^EQ-5D: EurolQol–5 Dimension.

^g^WAI-SF: Working Alliance Inventory-Short Form.

## Discussion

### Principal Findings

This study examined the feasibility of videoconference-delivered CBT in patients with bulimia nervosa and binge-eating disorder. Interventions based on CBT for bulimia nervosa and binge-eating disorder were conducted, and symptom improvement and acceptance by patients were determined before and after the intervention. Our results suggest that ICBT via videoconference for treating bulimia nervosa and binge-eating disorder is feasible. This is the second study in to conduct ICBT via videoconference that we know of and the first to apply this treatment strategy in Japanese patients with bulimia nervosa and binge-eating disorder. Five out of 7 participants completed treatment, for a completion rate of 71%. The mean combined objective binge and purging episodes and mean frequency of objective binge episodes and purging episodes on the EDE 16D for the primary outcome decreased: combined objective binge and purging episodes were reduced from 47.0 to 13.6 (71% reduction) with a moderate Cohen *d* of –0.76, objective binge episodes were reduced from 23.6 to 6.8 (71% reduction) with a large Cohen *d* of –0.82, and purging episodes were reduced from 23.4 to 6.8 (71% reduction) with a moderate Cohen *d* of –0.70, but no change was statistically significant. The abstinence rate was 43% (3/7). There were no significant treatment-related changes in any of the secondary outcomes. No adverse events were reported. Hence, our findings revealed that ICBT via videoconference for patients with bulimia nervosa and binge-eating disorder is feasible.

### Comparison With Previous Work

The results of this research are consistent with a previous RCT in the United States that used ICBT via videoconference and reported reductions in combined objective binge and purging episodes, objective binge episodes, and purging episodes (69%, 68%, and 70%, respectively) [18]. Our previous study with face-to-face CBT reported reduced combined objective binge and purging episodes, objective binge episodes, and purging episodes (51%, 50%, and 52%, respectively) [8]. This study has resulted in changes similar to our previous study. Furthermore, of the 5 people who completed treatment in this study, the abstinence rate was 60%. The abstinence rate among completers of ICBT via videoconference for bulimia nervosa and EDNOS was 37% [18]; the abstinence rate among face-to-face CBT for bulimia nervosa in our previous study was 40% [8]. Our results are better than previous studies about abstinence rate. Although previous studies did not report on effect size of combined objective binge and purging episodes [14,18], ICBT via videoconference had a moderate Cohen *d* of –0.66 for binging and a large Cohen *d* of –0.92 for purging at posttreatment, in our calculation [18]. According to a systematic review of ICBT, the range of Hedges effect sizes ranged from 0.75 to 1.05 for binge episodes and from 0.41 to 0.77 for purging episodes [14], which is similar to our findings. Furthermore, the results of our findings are similar to those of our previous study on CBT as a self-help strategy in Japanese patients with bulimia nervosa, in which the effect size of Cohen *d* for objective binge days (EDE 16D) was large, at 0.88, and for purging days was medium, at 0.67 [8]. Therefore, we believe that ICBT via videoconference is also feasible for Japanese patients with an eating disorder such as bulimia nervosa or binge-eating disorder.

First, for eating concern, a subscale of the EDE-Q, the effect size was large, although no significant difference was found in any of the secondary outcomes. Similar large effect sizes were observed for BITE severity and symptoms, which have been reported in previous studies [9,22]. Further, the BITE severity score (mean 4.6 [SD 3.4]) and symptoms score (mean 12.0 [SD 6.6]) were below the cutoff points of 5 and 20, respectively. The eating concern subscale assesses preoccupation with food, eating, or calories; fear of losing control over eating; eating in secret; social eating; and guilt about eating [26]. BITE, which is also related to the symptoms of overeating, had a similarly large effect size. Therefore, ICBT via videoconference may be promising for the treatment of overeating behavior and symptoms in patients with an eating disorder. However, there were no significant reductions in the restraint, shape concern, and weight concern subscales of the EDE-Q after treatment, and the effect size of Cohen *d* was small. A previous RCT in the United States using ICBT via videoconference reported a large effect size of Cohen *d* for these subscales of EDE-Q, in our calculation [18]. Similarly, our previous study on CBT as a self-help strategy in Japanese patients with bulimia nervosa reported a large effect size of Cohen *d* for these subscales of EDE-Q [8]. The effect of ICBT via videoconference on restraint, shape concern, and weight concern subscales of the EDE-Q may be relatively small in comparison with previous studies. Hence, there was no effect on dissatisfaction with body shape, aspiration for weight loss, or cognition to suppress food intake.

Second, the PHQ-9, which was used to evaluate symptoms of depression, had a small effect size, while the GAD-7, which was used to evaluate symptoms of anxiety, had a medium effect size. The mean GAD-7 score after treatment was below the cutoff value and decreased to a mild level. However, in our previous study of face-to-face CBT using the same treatment protocol, the effect sizes for PHQ-9 and GAD-7 were large [8]. The effect of ICBT via videoconference on symptoms of depression and anxiety associated with bulimia nervosa and binge-eating disorder may be relatively small in comparison with face-to-face CBT.

Third, the WAI-SF score in the range of 65.60 to 66.00 between pretreatment and posttreatment indicates that the patient’s relationship with the therapist was at a consistently high level during the intervention. In a previous study in the United States that analyzed WAI scores in patients with bulimia nervosa or EDNOS who received ICBT via videoconference, there was no significant difference in WAI scores between those who received face-to-face CBT and those who received ICBT via videoconference; the WAI scores for ICBT via videoconference indicated that this treatment was also highly rated [40]. Furthermore, motivation ruler scores were also consistently high (16.4 to 16.0) during the intervention. This is consistent with the result of cognitive remediation therapy for anorexia nervosa (motivation ruler scores 14.3 to 16.1) [41]. Even if the therapist and patient did not participate in a session in the same room, it was possible to maintain an adequately high level of motivation and a satisfactory therapeutic alliance via videoconference. We confirmed for the first time that therapeutic alliance between the patient and therapist via videoconference can be sufficiently developed among Japanese patients with bulimia nervosa and binge-eating disorder. Hence, it is feasible to use ICBT via videoconference for patients with eating disorder who live in remote areas without them physically visiting a hospital.

Finally, the dropout rate was 29% (2/7) in this study. An RCT of ICBT via videoconference for patients with bulimia nervosa and EDNOS reported a dropout rate of 34% (21/64) [18]. A poor treatment alliance and complications arising from comorbid mental illness such as depression may affect the dropout rate or treatment response [42,43]. The motivation score in patients who dropped out was relatively high (participant 6 had a score of 15 out of 20; participant 7 had a score of 20 out of 20), suggesting that motivation is not a reason for discontinuing CBT. According to previous research, the likelihood of discontinuation increases in subjects with less education, more novelty-seeking behavior, previous experience with CBT, and a mismatch between the preferred treatment and that assigned [44]. According to a meta-analysis of dropout rates in studies that included face-to-face CBT in patients with eating disorders, the diagnostic entity, definition of dropout, symptom severity at baseline, quality of the study, and patient age had no effect on the likelihood of discontinuation of CBT; the estimated overall dropout rate was 24% [45], which is similar to the study we are reporting. In our previous face-to-face CBT study, the treatment dropout rate was very low at 8% (2/25) [8]. It is interesting to note that despite the ICBT via videoconference being conducted from the same treatment manual, the study we are reporting now had a dropout rate of 29%. Although this appears to be a factor in ICBT, our previous study of patients receiving ICBT via videoconference for anxiety and obsessive-compulsive disorder reported a dropout rate of only 3% (1/30) [46], suggesting that the likelihood of discontinuation may be disease-specific rather than treatment-related. The dropout rate in patients receiving ICBT via videoconference for an eating disorder may be higher than that in their counterparts receiving face-to-face CBT. The patients who dropped out of this study (one with bulimia nervosa and one with binge-eating disorder) had comorbidities that required treatment with oral psychotropic medication in both cases. One patient had schizophrenia, dysthymia, and social anxiety disorder, and the other had posttraumatic stress disorder and generalized anxiety disorder. Eating disorders are often combined with significant impairment of social function and other psychiatric disorders including depression, anxiety, obsessive-compulsive disorder, and substance abuse disorder [47-49]. The distribution of such complications in the patients in this study can be considered representative of patients with eating disorders.

No adverse events were reported, suggesting that ICBT via videoconference is safe for patients with bulimia nervosa, as per a previous study [18]. Therefore, ICBT via video conference can be safely performed in Japanese patients with bulimia nervosa and binge-eating disorder.

### Limitations

There are some limitations in this study. The first is the small sample size, which meant that we could not determine whether our findings are due to a type II (β) error. G-power analysis was used to calculate the effect size, as described previously [9], which estimated the requirement of 15 samples. The second limitation is that the study was designed as a single-arm trial with no control group. To obtain statistically robust results, a clinical RCT is required that will estimate the sample size from the effect size of this study and will investigate the effectiveness of ICBT via videoconference to treat patients with bulimia nervosa and binge-eating disorder. We are presently planning such a trial in patients with eating disorders in Japan [UMIN000036825].

### Conclusions

Our results suggest that ICBT via videoconference for treating bulimia nervosa and binge-eating disorder is feasible. To our knowledge, this study is the world’s second clinical study of ICBT via videoconference and is the first to apply this treatment strategy in Japanese patients with bulimia nervosa and binge-eating disorder. The frequency of objective binge and purging episodes on the EDE 16D was reduced after treatment (71%). Hence, ICBT via videoconference has the potential to be an effective treatment in Japanese patients with bulimia nervosa and binge-eating disorder. The lack of any adverse events suggests that ICBT via videoconference is safe for bulimia nervosa and binge-eating disorder. RCTs are now needed to examine the effectiveness of this treatment strategy.

## Abbreviations

BITE: Bulimic Investigatory Test, Edinburgh
CBT: cognitive behavioral therapy
CONSORT-EHEALTH: Consolidated Standards of Reporting Trials of Electronic and Mobile Health Applications and Online TeleHealth
DSM-5: Diagnostic and Statistical Manual of Mental Disorders, 5th Edition
EDE 16D: Eating Disorder Examination Edition 16.0D
EDE-Q: Eating Disorders Examination Questionnaire
EDNOS: eating disorders not otherwise specified
EQ-5D-5L: EuroQol–5 Dimensions
GAD-7: 7-item Generalized Anxiety Disorder scale
ICBT: internet-based cognitive behavioral therapy
PHQ-9: 9-item Patient Heallth Questionnaire
RCT: randomized controlled trials
WAI-SF: Working Alliance Inventory–Short Form
